# Which institutional investors can improve the level of corporate ESG information disclosure?

**DOI:** 10.1371/journal.pone.0290008

**Published:** 2023-11-17

**Authors:** Jianye Wang, Yubing Ke, Huixue Zhang, Yusi Cheng

**Affiliations:** 1 School of Accounting, Guangdong University of Foreign Studies, Guangzhou, China; 2 School of Information Science and Technology, Guangdong University of Foreign Studies, Guangzhou, China; 3 Institute of Free Trade Zones, Sun Yat-sen University, Guangzhou, China; 4 School of Accounting, Guangdong University of Finance & Economics, Guangzhou, China; 5 School of Management Engineering, Zhengzhou University, Zhengzhou, China; University of Almeria: Universidad de Almeria, SPAIN

## Abstract

The inconsistency of existing findings on the relationship between institutional investors’ shareholdings and the level of corporate Environmental, Social and Governance (ESG) disclosure may lie in the insufficient consideration of the heterogeneity of institutional investors and investee firms. In this paper, from the perspective of institutional investor heterogeneity, we use a two-way fixed effects model to examine the impact of institutional investors on corporate ESG disclosure and the possible mechanism of this impact using a sample of Chinese A-share-listed firms from 2012 to 2020. We show that institutional investor shareholding can improve the level of corporate ESG information disclosure by enhancing auditor supervision and analyst attention to these external supervision. In terms of institutional investor heterogeneity, it is found that independent institutional investors and stable institutional investors play a stronger role in promoting the level of ESG information disclosure. Moreover, the positive net effect of the institutional investors on improving the level of ESG information disclosure is more pronounced in non-heavily polluting industries and state-owned enterprises. This paper enriches the impact of institutional investors’ shareholding on corporate ESG disclosure from a heterogeneity perspective.

## 1. Introduction

The influence of institutional investors on corporate Environmental, Social and Governance (ESG) information disclosure is a hot topic for scholars. However, existing studies have not reached a consistent conclusion on whether institutional investor shareholding can improve the ESG information disclosure level of invested enterprises. One view is that the shareholding of institutional investors can improve the level of ESG information disclosure of invested enterprises [1–3]. In this view, as an important participant in the capital market, institutional investors have professional knowledge and information advantages, motivation and ability to analyze the impact of macro policies [4], and their high shareholding ratio enables them to play the role of external supervision and internal governance in the invested enterprises [5]. So as to improve the level of ESG information disclosure of the invested enterprises. However, another viewpoint, from stakeholder salience perspective, believes that institutional investors pay more attention to the negative impact of ESG information disclosure process [6], thus inhibiting the level of ESG information disclosure of the invested enterprises. The third viewpoint holds that the shareholding of institutional investors has no effect on the ESG information disclosure of the invested enterprises [7, 8].

The possible reason for this result lies in insufficient consideration of the heterogeneity of institutional investors and investee enterprises [2]. Different institutional investors, due to their differences in investment ability and motivation, can bring different capital and supervision incentives to the invested enterprises, thus affecting the ESG information disclosure of the invested enterprises. For example, compared with independent institutional investors, non-independent institutional investors may pay more attention to short-term interests, and it is difficult for them to play a supervisory and governance role [9], and have a limited impact on corporate ESG information disclosure. Another example is the shareholding stability of institutional investors. The improvement of shareholding stability increases the enthusiasm of institutional investors to participate in corporate governance and supervision [10], which will further improve the level of corporate ESG information disclosure. The nature and characteristics of property rights of different enterprises lead to great differences in corporate governance, information disclosure and other aspects [11], as well as the supervisory influence that institutional investors can exert.

This paper adopts Chinese A-share listed companies from 2012 to 2020 as a sample and examines the impact of institutional investors on corporate ESG information disclosure and its possible mechanisms. The main contributions of this paper are as follows: First, from the perspective of institutional investor heterogeneity and corporate heterogeneity, the impact of institutional investor shareholding on corporate ESG information disclosure is enriched. This, to a certain extent, explains the inconsistence of existing research conclusions on the relationship between institutional investors and corporate ESG information disclosure [2]. Second, it enriches the research on mechanism of institutional investors’ influence on corporate ESG information disclosure. Existing studies mainly focus on the relationship between institutional investors and corporate ESG information disclosure, but lack of research on the influencing mechanism. Due to their high shareholding ratio, institutional investors can play a role of external supervision. This paper finds that institutional investors can improve the level of corporate ESG information disclosure by improving auditor supervision and analysts’ attention to such external supervision. Thirdly, various measurement tools and methods are used in this paper to eliminate the influence of endogeneity on the relationship between institutional investors and corporate ESG information disclosure to a certain extent. In this paper, instrumental variables and Heckman two-stage method are used, and the impact of environmental tax reform on the relationship between the two is considered, etc., to eliminate the influence of policy guidance and other factors as far as possible, so as to make the conclusion of the relationship between the two more robust.

The rest of this paper is organized, as follows. Section 2 outlines the theoretical analysis and research hypotheses. Section 3 describes the research method, while the empirical results and analysis are presented in Section 4. Section 5 presents further research and Section 6 offers the conclusions of this paper.

## 2. Theoretical analysis and research hypotheses

The existing literature infers that institutional investors have capital, professional and informational advantages and can influence the invested enterprises. On the one hand, institutional investors have more professional knowledge, stronger research teams and stronger information collection and analysis capabilities [12], which can better supervise invested enterprises. Furthermore, the higher shareholding ratio of institutional investors can not only make their supervision benefits more than their costs [13] but also enable them to have more rights to vote and elect directors. Thus, they have more motivation and better abilities to participate in and influence the decisions of invested enterprises. In addition, the withdrawal threat of institutional investors can also play a supervisory role [5]. Therefore, the shareholding of institutional investors can influence the investment behavior of invested enterprises and thus the results of their ESG information disclosure [14].

There are two different views of institutional investors on the influence of invested enterprises, one is value investment view and the other is speculation view [15].

The value investment theory holds that institutional investors pay attention to the long-term development of the invested enterprise, which represents a responsible investment behaviour [16]. Institutional investors hope to bring sustainable and effective financial and non-financial value-added effects to the invested enterprises by adhering to the concept of value investment and pursuing their interests [17]. Considering this, the impact of institutional investor shareholding on the ESG information disclosure of invested enterprises is mainly shown as follows. Based on carbon peak and carbon neutrality goal, institutional investors are motivated to improve enterprise ESG information disclosure levels. Excellent ESG disclosure quality will bring various resources to the enterprise, which can improve the enterprise value [18], thus improving institutional investors’ investment income. Therefore, institutional investors are motivated to work hard to improve the level of enterprise ESG information disclosure [19]. Moreover, institutional investors’ ability to improve the level of enterprise ESG information disclosure mainly has following four aspects. First, institutional investors have strong information advantages, which enable them to analyse the role of ESG in promoting the value of invested enterprises under the guidance of the ‘dual carbon’ policy [20], and then attach importance to the enterprise’s ESG performance in their investment and governance [21]. Second, the industry knowledge, management experience, social network and sensitivity to policies accumulated by institutional investors can guide and help the invested enterprises to pay more attention to ESG, increase ESG investment and improve the level of ESG information disclosure. In particular, institutional investors with a high proportion of shares in the invested enterprise often have seats on the board of directors and are more qualified to influence the ESG information disclosure of the enterprise by participating in the decision-making of the invested enterprise. Third, institutional investor shareholding can, to some extent, inhibit the behaviour of large shareholders’ joint management to hide negative ESG information about the enterprise for personal gain [2]. Thus, institutional investor shareholding improves the invested enterprise’s governance and the quality of ESG information disclosure of the enterprise. Finally, institutional investors can improve the quality of ESG information disclosure by improving the transparency of enterprise information [22]. Institutional investors have a professional analysis team and can dig out specific information about the invested enterprise through research and other behaviours and pass it on through the investor information network to improve the transparency of enterprise information [23], thereby improving the level of ESG information disclosure.

Another view, however, is that institutional investors are short-term investors who are short-sighted and speculative and hope to exit quickly and gain value-added income. They focus on their reputation and interests and do not consider the long-term development of the invested enterprise, which is contrary to the original intention of the value investment concept. ESG is an indicator that integrates environmental responsibility, social responsibility and corporate governance. It emphasizes that enterprises should pay attention to non-financial behaviours such as environmental protection, green production, corporate social responsibility, employee rights and interests protection, as well as corporate governance when pursuing maximum benefits. However, none of these goals can be achieved overnight. Enterprise ESG behavior is a long-term process. From a long-term perspective, the pursuit of ESG is conducive to the sustainable development of enterprises and increases enterprise value, which is consistent with the long-term development philosophy of institutional investors. In the short term, ESG behaviour will consume the capital, workforce and material resources of the enterprise and may even have little effect, which may not be consistent with the profit-seeking nature of institutional investors. Institutional investors with short-sighted and speculative ideas will reduce the level of ESG information disclosure. Institutional investors are not active stakeholders [24], and they may be short-sighted and speculative [25]. However, the ESG behaviour of enterprises is a long-term process that requires a long time [26], more capital and significant human and material resources, as such, the effect may not be obvious in the short term. The profit-seeking nature of institutional investors contradicts the ESG behaviour of enterprises. Institutional investors expect to exit from the investment quickly to realize value-added benefits. Therefore, when they intervene in the management decisions of the invested enterprises, they will intervene in the environmental protection, social responsibility and corporate governance investments that involve the long-term development of enterprises but affect short-term benefits [6], which may result in irregular waste disposal and insufficient social responsibility. Serious phenomena, such as the lack of protection of employees’ rights and interests, as well as the imbalance of corporate governance structure, hinder the sustainable development of enterprises and violate the core concept of ESG, indicating it as being poor enterprise ESG information disclosure.

Based on the above theoretical analysis, this paper proposes the following competitive assumptions:

H1a: The ESG information disclosure quality of enterprises held by institutional investors is better.
H1b: The ESG information disclosure quality of enterprises held by institutional investors is worse.

Due to differences in investment preference and investment philosophy, different types of institutional investors will have different degrees of supervision and incentive on the management, so their influence on the ESG information disclosure of the invested enterprises is also different. Therefore, it is necessary to distinguish the types of institutional investors and study their impact on ESG information disclosure of investee enterprises from the perspective of institutional investor heterogeneity [27]. Independent institutional investors and stable institutional investors adhere to the value investment concept and pursue long-term returns, so they have enough motivation to supervise and motivate the management, implement effective supervision on enterprises [28], improve the level of corporate governance, and further enhance the level of corporate ESG information disclosure. On the other hand, non-independent institutional investors and transactional institutional investors often have some kind of commercial connection or speculative motive with the invested enterprises, pay more attention to short-term interests, and are generally unwilling to destroy the short-term good relationship with enterprises, and lack motivation in urging enterprises to fulfill ESG responsibilities. Therefore, it is difficult to form effective supervision on enterprises [29]. Based on the above analysis, hypothesis H2a and H2b are proposed in this paper:

H2a: Compared with non-independent institutional investors, the ESG information disclosure quality of enterprises held by independent institutional investors is better.
H2b: Compared with transactional institutional investors, the ESG information disclosure quality of enterprises held by stable institutional investors is better.

Considering that in the capital market with Chinese characteristics, listed enterprises have two property rights, state-owned and non-state-owned, and the difference in property rights makes enterprises differ greatly in corporate governance, information disclosure and other aspects [30]. The business objectives of state-owned enterprises have both economic and social attributes, so they need to undertake extensive ESG responsibilities. Institutional investors improve ESG information disclosure by participating in corporate governance, which is intrinsically consistent with non-economic objectives of state-owned enterprises. Therefore, institutional investors may play a more effective supervisory role in ESG information disclosure of state-owned enterprises.

In terms of corporate pollution attributes, due to the implementation of China’s "two-carbon" policy, enterprises in heavily polluting industries have received more social attention, especially facing greater regulatory pressure from the government, which requires more disclosure of ESG information [11]. Therefore, the level of ESG information disclosure may be relatively high. The role of institutional investors in improving the level of ESG information disclosure is relatively weak.

Based on the above analysis, hypothesis H3a and H3b are proposed in this paper:

H3a: Compared with non-state-owned enterprises, state-owned enterprises held by institutional investors have better ESG information disclosure quality.
H2b: Compared with enterprises in heavy polluting industries, the ESG information disclosure quality of enterprises in non-heavy polluting industries held by institutional investors is better.

## 3. Research methods

### 3.1 Sample selection and data source

This paper took Chinese A-share listed enterprises from 2012 to 2020 as the initial research samples and selected them according to the following criteria: (1) ST and *ST enterprises were excluded; (2) Listed enterprises in the financial industry were excluded; (3) Samples with missing or incomplete data were removed. This resulted in 10,749 observations. ESG data derived from the overall ESG scores and rates of listed enterprises on Hexun website. The data relating to enterprise finance was derived from China Stock Market and Accounting Research database (CSMAR). To avoid the influence of outliers, continuous variables were curtailed at 1% and 99% levels.

### 3.2 Variable definitions

The dependent variable is the quality of enterprise ESG information disclosure, specifically divided into enterprise ESG score (ESG-S) and enterprise ESG rating (ESG-R). The quality of enterprise ESG information disclosure is measured by Hexun’s ESG evaluation system for listed enterprises. Hexun’s evaluation of the ESG of listed enterprises is based on 97 aspects of environmental responsibility, social responsibility and corporate governance, according to which an overall score and rating is derived. The total score of the ESG is 100, and the higher the score, the better the enterprise’s ESG performance. The ESG rating is divided into five grades as follows: A is the highest grade with a value of 5, and E is the lowest grade with a value of 1.

The independent variable is the shareholding ratio of institutional investors (INS).

The control variables include enterprise size (Size), enterprise age (Age), asset-liability ratio (Lev), growth opportunity (Growth), enterprise performance (ROA), price-earnings ratio (PE), the shareholding ratio of the largest shareholder (Ratio) and the independence of board of directors (Indepe). The specific definitions of all variables are shown in Table 1.

**Table 1:** Variable definitions.

Variable Type	Variable Name	Variable Symbolic	Variable Definitions
Dependent variables	ESG score	ESG-S	The overall score of Hexun website on the enterprise’s ESG performance is 100
	ESG rating	ESG-R	The highest rating of enterprise ESG by the Hexun website is 5 for Level A and 1 for Level E
Independent variables	The shareholding ratio of institutional investors	INS	The proportion of shares held by institutional investors in total share capital at the end of the year
Control variables	enterprise size	Size	Natural logarithm of total assets at the end of the period
	enterprise age	Age	Number of years of listing
	asset-liability ratio	Lev	Total liabilities at the end of the period/total assets at the end of the period
	growth opportunity	Growth	The growth rate of business income
	enterprise performance	ROA	Return on capital invested by enterprises
	price-earnings ratio	PE	Market price per share/earnings per share
	the shareholding ratio of the largest shareholder	Ratio	Number of shares held by the largest shareholder/total number of shares
	independence of the board of directors	Indepe	Number of independent directors/total number of directors

### 3.3 Model

To verify the hypotheses, we set the following regression model. We use the two-way fixed effect model for regression and perform a robust treatment on the standard error of the regression results to correct the heteroscedasticity problem.


$${ESG}_{i,t}=\beta_{0}+\beta_{1}{INS}_{i,t}+\beta_{2}{Controls}_{i,t}+\mu_{i}+\eta_{t}+\varepsilon_{i,t}$$ (1)

In the formula, i represents the enterprise, t represents the year, ESG represents the enterprise’s quality of ESG information disclosure, and ESG-S and ESG-R of the enterprise in the ith year are used, respectively. Further, INS represents institutional investors’ shareholding, and Controls represent control variables, *μ_i_* refers to the fixed effect of individual enterprises and *η_t_* refers to the annual fixed effect.

## 4. Empirical results and analysis

### 4.1 Descriptive statistics

Table 2 shows the descriptive statistical results of the whole sample. The mean value of the dependent variable ESG-S is 24.490, and the standard deviation is 17.037. Further, the minimum value is -4.810, the median is 21.850 and the maximum is 76.040. The mean value of ESG-R is 1.785, the standard deviation is 0.868, the minimum value is 1, the median value is 2 and the maximum value is 5. To sum up, the quality of ESG information disclosure of Chinese listed enterprises is generally low, most of which are rated as Grade D. Thus, the level of ESG information disclosure needs further improvement. The mean value of the independent variable institutional investors shareholding ratio (INS) is 0.463, and its standard deviation is 0.227, indicating that most enterprises have institutional investors and the shareholding ratio is maintained within a specific range. The higher shareholding ratio of institutional investors indicates that they have the conditions to influence the invested enterprises.

**Table 2:** Descriptive statistics of the whole sample.

Variables	Sample size	Mean	Standard deviation	Min	Median	Max
ESG-S	10749	24.490	17.037	-4.810	21.850	76.040
ESG-R	10749	1.785	0.868	1.000	2.000	5.000
INS	10749	0.463	0.227	0.004	0.479	0.908
Size	10749	22.293	1.276	19.383	22.145	25.990
Age	10749	14.299	6.551	2.481	14.433	27.117
Lev	10749	0.372	0.223	0.002	0.359	0.916
Growth	10749	0.233	1.428	-0.964	0.000	11.695
ROA	10749	0.030	0.063	-0.280	0.029	0.194
PE	10749	70.664	141.817	0.000	28.160	1005.488
Radio	10749	0.335	0.149	0.081	0.310	0.740
Indepe	10749	0.262	0.177	0.000	0.333	0.571

### 4.2 Analysis of the regression results

Table 3 shows the regression results. The dependent variable of columns (1) and (2) is the overall ESG-S of the enterprise, and the dependent variable of columns (3) and (4) is the overall ESG-R of the enterprise. Columns (1) and (3) do not include control variables. It can be seen that the coefficient of institutional investors’ shareholding is significantly positive at the level of 1%, which preliminarily verifies the hypothesis H1a. After adding control variables to columns (2) and (4), it is found that the coefficients of institutional investors’ shareholding are 0.023 and 0.002, respectively, which are significantly positive at the levels of 5% and 1%. This indicates that every 1% increase in institutional investors’ shareholding can promote the quality of enterprise ESG information disclosure by 0.023% and 0.002%. Therefore, the results further verify the hypothesis H1a. This shows that institutional investors are pursuing long-term interests. They believe that the quality of ESG information disclosure of enterprises is consistent with their investment philosophy. When participating in enterprise decision-making, they will actively urge and promote enterprises to implement ESG behaviour, thus improving the level of ESG information disclosure. Among the control variables, the coefficients of Size and Age are both significantly positive at 1%, indicating that the quality of ESG information disclosure of enterprises with large scale and an early establishment is better. ROA coefficients are significantly positive, indicating that enterprises with a higher return on asset investment have better fund use effect and can show better ESG information disclosure quality.

**Table 3:** Institutional investor shareholding and ESG information disclosure.

Variables	(1)	(2)	(3)	(4)
	ESG-S	ESG-S	ESG-R	ESG-R
INS	0.151*** (21.030)	0.023** (2.494)	0.008*** (21.012)	0.002*** (3.891)
Size		3.798*** (24.447)		0.196*** (23.286)
Age		0.078*** (2.914)		0.004** (2.401)
Lev		-0.238 (-0.259)		-0.046 (-0.929)
Growth		0.124 (1.209)		0. 006 (1.011)
ROA		102.722*** (40.297)		3.755*** (27.173)
PE		-0.004*** (-3.603)		-0.001*** (-11.190)
Ratio		-0.005 (-0.317)		-0.001 (-0.808)
Indepe		0.418 (1.200)		0.020 (1.074)
Firm Effect	Control	Control	Control	Control
Year Effect	Control	Control	Control	Control
N	10749	10749	10749	10749
Adjusted R-squared	0.222	0.406	0.196	0.331

Note: ***, * * and * represent significance levels of 1%, 5% and 10%, respectively. The figures in parentheses are t values, and the regression results were treated by Robust standard error.

### 4.3 Robustness testing

#### 4.3.1 Replacing variables

First, replace the dependent variables. The measurement method for the quality of ESG information disclosure of dependent variable enterprises is replaced by the Chinese Securities Index ESG rating for inspection. The highest value of AAA is 9, and the lowest value of C is 1. The results in Table 4 do not affect the conclusions of baseline regression.

**Table 4:** Robustness testing by replacing variables.

Variables	Replace the dependent variables		Replace the independent variables	
	ESG-R	ESG-R	ESG-S	ESG-R
INS	0.012*** (22.652)	0.004*** (5.006)	0.061** (2.009)	0.034** (2.104)
Size		0.393*** (31.225)	3.834*** (24.685)	0.198*** (23.506)
Age		0.020*** (9.659)	0.112*** (4.362)	0.005*** (3.709)
Lev		0.143* (1.922)	-0.075 (-0.081)	-0.038 (-0.768)
Growth		0.004 (0.417)	0.120 (1.174)	0.005 (0.979)
ROA		2.364*** (11.454)	102.22*** (40.098)	3.730*** (26.997)
PE		-0.000*** (-3.939)	-0.004*** (-3.476)	-0.001*** (-11.072)
Ratio		-0.002 (-1.373)	-0.009 (-0.554)	-0.001 (-1.024)
Indepe		-0.119*** (-4.243)	0.196 (0.569)	0.009 (0.500)
Firm Effect	Control	Control	Control	Control
Year Effect	Control	Control	Control	Control
N	10749	10749	10749	10749
Adjusted R-squared	0.161	0.280	0.191	0.133

Note: ***, * * and * represent significance levels of 1%, 5% and 10%, respectively. The figures in parentheses are t values, and the regression results were treated by Robust standard error.

Second, replace the independent variables. According to the median of institutional investors’ shareholding ratio, the dummy variable is constructed by grouping, and the regression is conducted again. The results in Table 4 show that institutional investors with higher shareholding ratios have better improved the ESG information disclosure level of the invested enterprise, which proves the robustness of the original regression results.

#### 4.3.2 Lag effects and environmental policies

First, consider the lag effects. Considering that institutional investors’ shareholding influence on the level of enterprise ESG information disclosure may have a lag effect, the dependent variable is regressed again after a lag period. The results in Table 5 do not affect the conclusions of the original regression, and the regression coefficients improve significantly.

**Table 5:** Robustness testing by lag effects and environmental policies.

Variables	Dependent variable lags by one period		Consider environmental policies	
	ESG-S	ESG-R	Before 2017	After 2017
			ESG-R	ESG-R
INS	0.043*** (4.355)	0.003*** (4.715)	0.002*** (2.722)	0.002*** (4.145)
Size	3.418*** (18.944)	0.167*** (17.499)	0.270*** (16.036)	0.074*** (10.929)
Age	0.096*** (3.263)	0.004** (2.556)	0.011*** (4.056)	-0.000 (-0.182)
Lev	0.363 (0.343)	-0.023 (-0.418)	0.022 (0.221)	-0.049 (-1.259)
Growth	0.125 (1.078)	0.002 (0.329)	-0.003 (-0.258)	0.007* (1.785)
ROA	51.972*** (17.226)	2.812*** (17.676)	6.199*** (18.391)	2.322*** (26.491)
PE	-0.014*** (-11.033)	-0.001*** (-10.233)	-0.000*** (-4.380)	-0.001*** (-12.961)
Ratio	0.001 (0.030)	-0.000 (-0.422)	-0.001 (-0.813)	0.002** (2.344)
Indepe	-0.060 (-0.154)	0.004 (0.208)	0.033 (0.901)	0.014 (0.889)
Firm Effect	Control	Control	Control	Control
Year Effect	Control	Control	Control	Control
N	10749	10749	4316	5130
Adjusted R-squared	0.334	0.298	0.282	0.373

Note: ***, * * and * represent significance levels of 1%, 5% and 10%, respectively. The figures in parentheses are t values, and the regression results were treated by Robust standard error.

Second, consider the impact of environmental protection policies. The Environmental Protection Tax Law of the People’s Republic of China was introduced on December 25, 2016, changing environmental protection fees into taxes. This policy has significantly improved the level of enterprise ESG related information disclosure [31]. Therefore, we consider whether the impact of institutional investors on the level of enterprise ESG information disclosure is due to the introduction of this policy. According to the Environmental Protection Tax Law of the People’s Republic of China, 2017 is considered as a separate time axis, and the span of control years is the same. We take 2013–2016 as the period before the policy was issued and 2017–2020 as the period after the policy was issued, so the number of samples is reduced to 9,446. The regression results in Table 5 show that the original regression conclusions remain unchanged. However, compared with the original regression, the impact of institutional investors on the enterprise’s ESG information disclosure level significantly decreased before the introduction of the policy. However, the increasing significance of this influence after the introduction of the policy indicates that the influence of institutional investors’ shareholding on corporate ESG information disclosure is not caused by environmental protection policies, but environmental protection policies will strengthen the influence of institutional investors on corporate ESG information disclosure.

### 4.4 Endogeneity treatment

First, we consider the tool variable method. Referring to the paper of Liang [32], we use one dummy as the instrumental variable of institutional investors shareholding ratio. This dummy is whether the company belongs to the top 300 in Shenzhen Stock Exchange and Shanghai Stock Exchange (HS300) or not. When the company is among the top 300 in Shenzhen Stock Exchange and Shanghai Stock Exchange, the HS300 value is 1; otherwise, it is 0. HS300 is selected as the tool variable of institutional investors shareholding because the Shanghai and Shenzhen 300 Index (CSI300) of China stock market has good fundamentals, is widely concerned by the market, and is closely related to the shareholding ratio of institutional investors. At the same time, it has no direct relationship with enterprises’ future ESG information disclosure quality. The regression results in Table 6 show that HS300 and INS are significantly positively correlated at a 1% level in the first stage, while the original regression results do not change significantly in the second stage.

**Table 6:** Endogeneity treatment results.

Variables	Tool variable method			Heckman two-stage method		
	INS	ESG-S	ESG-R	INS	ESG-S	ESG-R
INS		0.034*** (3.287)	0.002*** (4.254)		0.034*** (3.812)	0.003*** (5.133)
HS300	0.056*** (4.144)			0.139** (2.321)		
IMR					1.887*** (4.003)	0.094*** (3.669)
Size	0.382*** (17.191)	3.256*** (16.478)	0.189*** (14.521)		3.064*** (12.401)	0.160*** (11.919)
Age	0.053*** (16.005)	0.025*** (2.964)	0.003** (2.437)		0.039 (1.221)	0.002 (0.884)
Lev	-1.170*** (-8.897)	0.286 (0.489)	-0.053 (-0.756)		2.214** (2.042)	0.075 (1.282)
Growth	-0.023* (-1.657)	0.187 (1.112)	0.002 (0.458)		0.163 (1.584)	0.008 (1.355)
ROA	2.510*** (7.220)	34.285*** (12.332)	2.563*** (14.365)		97.458*** (34.669)	3.494*** (22.927)
PE	-0.000 (-1.002)	-0.028*** (-8.986)	-0.002*** (-7.698)		-0.003*** (-3.237)	-0.001*** (-10.844)
Ratio	0.128*** (50.744)	0.002 (0.134)	-0.000 (-0.587)		-0.251*** (-4.007)	-0.013*** (-3.809)
Indepe	1.621*** (35.027)	-0.075 (-0.259)	0.005 (0.563)		-2.519*** (-3.311)	-0.126*** (-3.044)
Vcright				0.028*** (9.193)	-4.203*** (-3.776)	-0.333*** (-5.511)
Trans				0.035*** (4.819)	-1.296*** (-3.962)	-0.051*** (-2.855)
Inter-score				0.099** (2.361)	-0.292 (-0.912)	-0.020 (-1.159)
East				0.003 (0.563)		
Firm Effect	Control	Control	Control	Control	Control	Control
Year Effect	Control	Control	Control	Control	Control	Control
N	10749	10749	10749	10749	10749	10749
Adjusted R-squared	0.337	0.356	0.287	0.506	0.405	0.331

Note: ***, * * and * represent significance levels of 1%, 5% and 10%, respectively. The figures in parentheses are t values, and the regression results were treated by Robust standard error.

Second, we adopt Heckman’s two-stage method. Some unobservable characteristics of enterprises may affect the selection of enterprises by institutional investors and the level of ESG information disclosure of enterprises simultaneously, leading to selective bias in the sample. This paper uses Heckman’s two-stage method to modify the possible endogeneity. In the first stage, a probit model that affects institutional investors’ shareholding is established to calculate the inverse Mills ratio (IMR). In the selection model, INS is the explained variable. If it exceeds the median, the value is 1; otherwise, it is 0. There are five independent variables. The first one is whether the company belongs to the HS300. The second one is the difference between the voting rights proportion and the cash flow rights proportion (Vcright), which is the proportion of control rights of listed enterprises owned by the actual controller minus the proportion of ownership held by them. The third one is information transparency (Trans), measured by the sum of the absolute values of the enterprise’s manipulative accruals in the previous three years. The internal control level (Inter score) is measured by the natural logarithm of the enterprise’s internal control index plus 1. The last one is the geographic location (East). If the enterprise is located in the eastern province, the geographic location value takes 1; otherwise, it takes 0. In the second stage, the inverse Mills ratio is added to the original regression model as a control variable for regression. The results in Table 6 show that IMR is significant at the 1% level, indicating that sample selection bias exist. However, the impact of institutional investors’ shareholding ratio on the enterprise’s ESG information disclosure level is still significantly positive at the 1% level, indicating that the original regression results are still valid after considering sample selection bias.

## 5. Further research

### 5.1 Mechanism testing

Institutional investors have professional and informational advantages. Their stock selections can transmit a value signal, attract the attention of securities analysts and other external supervisors, improve the level of external supervision of enterprises [33], and thus improve the quality of enterprise ESG information disclosure. This paper uses auditor supervision (Big4) and analysts’ concern (Forecast) to measure the external supervision of enterprises and analyses the mechanism of institutional investors affecting the level of enterprise ESG information disclosure. The Big4 is measured by whether it is audited by the four major accounting firms, and the analysts’ concern (Forecast) is measured by the logarithm of the number of analysts tracking the enterprise within one year plus one.

Table 7 shows the regression results of the mechanism testing. The results of columns (1) and (3) show that institutional investors shareholding can significantly improve auditor supervision and analysts’ attention at the level of 1%. Columns (2) and (4), respectively, add auditor supervision and analysts’ concern to the regression analysis of institutional investor shareholding on the enterprise ESG information disclosure level. The results show that auditor supervision and analysts’ concern can significantly improve the level of enterprise ESG information disclosure at the level of 1%, and the impact of institutional investors’ shareholding remains unchanged. The above results show that institutional investors can improve the level of ESG information disclosure by improving auditor supervision and analysts’ attention to these external supervisions.

**Table 7:** Mechanism testing results.

Variables	Auditor supervision		Analysts’ attention	
	(1) Big4	(2) ESG-S	(3) Forecast	(4) ESG-S
Big4		2.438*** (4.750)		
Forecast				0.123*** (7.412)
INS	0.001*** (6.766)	0.030*** (3.328)	0.085*** (16.401)	0.022** (2.444)
Size	0.089*** (30.022)	3.618*** (22.380)	4.972*** (55.065)	3.221*** (18.347)
Age	-0.002*** (-3.015)	0.116*** (4.503)	-0.301*** (-20.097)	0.150*** (5.708)
Lev	-0.049*** (-2.806)	0.044 (0.048)	-4.110*** (-7.669)	0.431 (0.468)
Growth	0.002 (0.946)	0.116 (1.132)	0.147** (2.470)	0.102 (1.000)
ROA	-0.102** (-2.130)	102.467*** (40.228)	47.355*** (31.952)	96.383*** (36.210)
PE	-0.000 (-1.113)	-0.004*** (-3.428)	-0.008*** (-12.611)	-0.003** (-2.560)
Ratio	0.001*** (3.015)	-0.012 (-0.693)	-0.150*** (-15.538)	0.009 (0.554)
Indepe	0.024*** (3.609)	0.139 (0.403)	-1.340*** (-6.682)	0.362 (1.048)
Firm Effect	Control	Control	Control	Control
Year Effect	Control	Control	Control	Control
N	10749	10749	10749	10749
Adjusted R-squared	0.227	0.406	0.477	0.408

Note: ***, * * and * represent significance levels of 1%, 5% and 10%, respectively. The figures in parentheses are t values, and the regression results were treated by Robust standard error.

### 5.2 Heterogeneity analysis

#### 5.2.1 Heterogeneity analysis of institutional investors

According to the paper of Chen et al. [34], we divide institutional investors into independent institutional investors and non-independent institutional investors. Independent institutional investors (INS-INDE) are institutional investors that have no business relationship with enterprises, including funds, social security and qualified foreign institutional investor (QFII). Non-independent institutional investors (INS-DE) refer to institutional investors other than independent institutional investors, which are generally considered to have certain business relationships with listed enterprises. As shown in Table 8 (1) and (2), INS-INDE significantly impact the quality of enterprise ESG information disclosure, while INS-DE have no significant impact. Hypothesis H2a is verified. These results are consistent with previous studies, indicating that INS-DE may pay more attention to short-term interests and are less likely to play a supervisory and governance role.

**Table 8:** Analysis of the heterogeneity of institutional investors.

Variables	(1) ESG-S		(2) ESG-R		(3) ESG-S	(4) ESG-R
INS					0.024** (2.340)	0.002*** (3.637)
INS-INDE	0.186*** (7.042)		0.015*** (10.191)			
INS-DE		0.006 (0.624)		0.001 (0.934)		
IIC					1.484** (1.973)	0.102** (2.495)
INS×IIC					0.029** (2.059)	0.002*** (2.475)
Size	3.712*** (24.135)	3.978*** (26.142)	0.188*** (22.556)	0.208*** (25.256)	3.735*** (23.729)	0.192*** (22.462)
Age	0.152*** (6.111)	0.133*** (5.102)	0.008*** (6.064)	0.007*** (4.722)	0.102*** (3.888)	0.005*** (3.301)
Lev	0.041 (0.044)	-0.373 (-0.405)	-0.028 (-0.561)	-0.060 (-1.204)	0.018 (0.019)	-0.028 (-0.560)
Growth	0.112 (1.098)	0.113 (1.105)	0.005 (0.876)	0.005 (0.885)	0.121 (1.200)	0.005 (0.983)
ROA	98.788*** (37.927)	102.959*** (40.482)	3.459*** (24.548)	3.786*** (27.445)	100.218*** (39.315)	3.627*** (26.204)
PE	-0.003*** (-3.016)	-0.004*** (-3.529)	-0.001*** (-10.420)	-0.001*** (-11.135)	-0.004*** (-3.589)	-0.001*** (-11.067)
Ratio	0.041*** (3.086)	0.021 (1.179)	0.003*** (3.971)	0.001 (1.304)	-0.008 (-0.473)	-0.001 (-1.169)
Indepe	0.786** (2.449)	0.568 (1.614)	0.054*** (3.085)	0.036* (1.900)	0.199 (0.561)	0.006 (0.312)
Firm Effect	Control	Control	Control	Control	Control	Control
Year Effect	Control	Control	Control	Control	Control	Control
N	10749	10749	10749	10749	10536	10536
Adjusted R-squared	0.407	0.404	0.335	0.328	0.408	0.322

Note: ***, * * and * represent significance levels of 1%, 5% and 10%, respectively. The figures in parentheses are t values, and the regression results were treated by Robust standard error.

Moreover, institutional investors are divided into stable and transactional institutional investors according to Yang et al. [35]. First, we calculate the shareholding ratio of institutional investors of enterprise i in year t and then divide it by the standard deviation (SD) of the shareholding ratio in the past three years to obtain the ratio. Second, if the SD of enterprise i is greater than the median level of the same industry in the same year, its institutional investors are defined as stable institutional investors (IIC = 1); otherwise, they are defined as transactional institutional investors (IIC = 0). Then we add the variable ‘stable institutional investors’ (IIC), institutional investors’ shareholding and IIC multiplying items in the model. The regression results are shown in Table 8, columns (3) and (4). The higher the stability of institutional investors’ shareholding, the better the quality of institutional investors’ shareholding on enterprise ESG information disclosure. This shows that the improvement of institutional investors’ shareholding stability increases the enthusiasm of institutional investors to participate in corporate governance and supervision. Hypothesis H2b is verified.

#### 5.2.2 Analysis of enterprise heterogeneity

Considering that in the capital market with Chinese characteristics, listed enterprises have two kinds of property rights, state-owned and non-state-owned, the difference in property rights makes enterprises differ greatly in corporate governance, information disclosure, etc. Institutional investors who enter state-owned enterprises (SOEs) as non-public shareholders may play a supervisory and governance role in the information disclosure of SOEs. This leads to a more obvious impact of institutional investors’ shareholding on the quality of ESG information disclosure of SOEs. This paper regresses the property right nature of the whole sample. The results are shown in Table 9. The regression coefficients of the sample group of SOEs is significantly positive at the level of 1%, while that of the sample group of non-state-owned enterprises (non-SOEs) is not significant. This shows that institutional investors’ shareholding plays a stronger role in improving the quality of ESG information disclosure of SOEs. Hypothesis H3a is verified.

**Table 9:** Heterogeneity analysis of enterprise property rights.

Variables	ESG-S		ESG-R	
	SOEs	Non-SOEs	SOEs	Non-SOEs
INS	0.130*** (5.165)	-0.001 (-0.074)	0.007*** (5.483)	0.001 (1.393)
Size	3.511*** (14.567)	3.481*** (16.342)	0.174*** (13.503)	0.188*** (15.980)
Age	0.056 (1.263)	0.109*** (3.066)	0.002 (0.762)	0.006*** (3.025)
Lev	-0.708 (-0.504)	0.485 (0.388)	-0.062 (-0.818)	-0.019 (-0.274)
Growth	0.324** (2.080)	-0.076 (-0.578)	0. 011 (1.344)	-0.001 (-0.160)
ROA	112.212*** (22.922)	98.081*** (34.405)	4.242*** (16.191)	3.487*** (22.165)
PE	-0.001 (-0.477)	-0.006*** (-4.323)	-0.001*** (-5.865)	-0.001*** (-9.527)
Ratio	-0.105*** (-3.012)	0.029 (1.442)	-0.006*** (-3.121)	0.001 (0.793)
Indepe	-1.951** (-2.452)	0.858** (2.192)	-0.097** (-2.286)	0.043** (1.971)
Firm Effect	Control	Control	Control	Control
Year Effect	Control	Control	Control	Control
N	4874	5875	4874	5875
Adjusted R-squared	0.428	0.410	0.371	0.314

Note: ***, * * and * represent significance levels of 1%, 5% and 10%, respectively. The figures in parentheses are t values, and the regression results were treated by Robust standard error.

Moreover, this paper divides Chinese listed enterprises into two groups according to whether they belong to heavy pollution industries and tests the difference in the impact of institutional investors’ shareholding on the quality of ESG information disclosure of these two types of enterprises. If the enterprise belongs to the mining, textile, clothing, fur, metallic and non-metallic, biological medicine, petrochemical plastic, paper and printing industry, water, electricity and gas, food and beverage industry, etc., it is defined as a heavily polluted enterprise; otherwise, it is defined as the non-heavily polluted enterprise. The regression results are shown in Table 10. The regression coefficients of the sample group of non-heavily polluted enterprises are significantly positive at the 1% level, while the sample group of heavily polluted enterprises are insignificant. This shows that institutional investors’ shareholding can only significantly promote the quality of ESG information disclosure in non-heavily polluting enterprises. Hypothesis H3b is verified. The possible reason is that enterprises in heavily polluted industries receive more social attention than non-heavily polluted enterprises, especially facing greater government regulatory pressure and the need to disclose more ESG information [36]. Therefore, the level of ESG information disclosure is relatively high, and the role of institutional investors in improving the level of ESG information disclosure is relatively weak.

**Table 10:** Heterogeneity analysis of industry differences.

Variables	ESG-S		ESG-R	
	Pollution	Non-Pollution	Pollution	Non-Pollution
INS	0.005 (0.376)	0.053*** (4.485)	0.001 (1.373)	0.004*** (5.479)
Size	3.758*** (14.596)	3.859*** (19.729)	0.194*** (13.863)	0.200*** (18.941)
Age	0.069 (1.624)	0.156*** (4.784)	0.002 (0.740)	0.008*** (4.575)
Lev	1.136 (0.692)	-0.661 (-0.596)	-0.016 (-0.181)	-0.050 (-0.836)
Growth	0.204 (1.168)	0.058 (0.463)	0.010 (1.014)	0.002 (0.301)
ROA	107.774*** (26.328)	97.801*** (30.015)	4.172*** (18.744)	3.375*** (19.168)
PE	-0.005*** (-3.055)	-0.003** (-1.990)	-0.001*** (-8.569)	-0.001*** (-7.174)
Ratio	0.033 (1.243)	-0.048** (-2.212)	0.001 (0.680)	-0.003** (-2.428)
Indepe	0.319 (0.573)	-0.012 (-0.028)	0.028 (0.941)	-0.011 (-0.463)
Firm Effect	Control	Control	Control	Control
Year Effect	Control	Control	Control	Control
N	4577	6172	4577	6172
Adjusted R-squared	0.377	0.429	0.312	0.349

Note: ***, * * and * represent significance levels of 1%, 5% and 10%, respectively. The figures in parentheses are t values, and the regression results were treated by Robust standard error.

## 6. Discussion and conclusions

This paper uses the Chinese A-share listed enterprises from 2012 to 2020 as the sample to study the relationship between institutional investors and enterprises’ ESG information disclosure. It starts with the external factors that affect the ESG information disclosure of enterprises and studies the impact and mechanism of institutional investors’ shareholding on the quality of enterprise ESG information disclosure. The empirical results show that institutional investor shareholding can significantly improve the level of ESG information disclosure of Chinese listed companies. The analysis of the impact mechanism shows that institutional investor shareholding improves the level of ESG information disclosure by upgrading auditor supervision and analysts’ attention to this external supervision. Heterogeneity analysis shows that independent institutional investors are more pronounced in improving the quality of ESG information disclosure. The higher the stability of institutional investor shareholding, the better the quality of ESG information disclosure. Institutional investor shareholding plays a strong role in improving the quality of ESG information disclosure of SOEs and enterprises in non-heavily polluting industries. The original conclusions are still robust after replacing variable measurements, considering the lag effect and environmental policy impact, and using tool variables and Heckman’s two-stage method to eliminate endogenous problems.

This paper enriches the impact of institutional investors’ shareholding on corporate ESG disclosure from a heterogeneity perspective. This explains, to some extent, the inconsistency of the findings of existing studies on the relationship between institutional investors and corporate ESG disclosure [2]. In addition, this paper further extends the research on the mechanism of institutional investors’ influence on corporate ESG disclosure.

This finding has important policy and academic implications. For investee companies, it is necessary to understand that different types of institutional investors may have different levels of incentives to supervise management due to differences in investment preferences and investment philosophies, etc., and therefore there are some differences in their impact on ESG disclosure of investee companies. Investee companies need to cooperate more with independent and stable institutional investors to improve the quality of ESG disclosure. For regulators, on the one hand, regulators should create conditions to encourage institutional investors to actively participate in corporate governance and supervision, and on the other hand, they also need to cultivate stable institutional investors, and give full play to their motivation and ability in enhancing the level of ESG disclosure of enterprises; in addition to this, in the mixed ownership reform of state-owned enterprises (SOEs), they can guide institutional investors to enter SOEs as non-public shareholders, and give full play to their role in SOEs’ ESG information disclosure. For researchers, the differences in heterogeneity of institutional investors need to be considered when studying the relationship between institutional investors and corporate ESG disclosure. This paper examines the impact of institutional investors on corporate ESG disclosure performance and draws relatively robust conclusions, but there are still some limitations, and future research can continue to expand in the following aspects. On the one hand, in addition to the external monitoring mechanisms of auditor supervision and analyst attention mentioned in this paper, subsequent research can continue to delve deeper into other relevant mechanisms of institutional investors affecting corporate ESG disclosure performance, such as the signing of betting agreements, if detailed information on investment covenants can be obtained. On the other hand, although the possible endogeneity problem is solved by applying the replacement variable measurement, considering the lagged effect and the impact of environmental policies as well as the use of instrumental variables and Heckman’s two-stage method after controlling for the financial characteristics of the firms, corporate governance and other characteristics. However, because of the lack of exogenous shock events, the interference of endogeneity problems cannot be completely ruled out, and the endogeneity problems can be better solved subsequently by finding more appropriate methods.

## Supporting information

S1 Dataset (XLSX)
